# RNase P: Beyond Precursor tRNA Processing

**DOI:** 10.1093/gpbjnl/qzae016

**Published:** 2024-02-12

**Authors:** Peipei Wang, Juntao Lin, Xiangyang Zheng, Xingzhi Xu

**Affiliations:** Guangdong Key Laboratory for Genome Stability & Disease Prevention and Marshall Laboratory of Biomedical Engineering, Shenzhen University Medical School, Shenzhen University, Shenzhen 518060, China; Guangdong Key Laboratory for Genome Stability & Disease Prevention and Marshall Laboratory of Biomedical Engineering, Shenzhen University Medical School, Shenzhen University, Shenzhen 518060, China; Department of Urology, The First Affiliated Hospital, Zhejiang University School of Medicine, Hangzhou 310000, China; Shenzhen University General Hospital-Dehua Hospital Joint Research Center on Precision Medicine, Dehua Hospital, Dehua 362500, China; Guangdong Key Laboratory for Genome Stability & Disease Prevention and Marshall Laboratory of Biomedical Engineering, Shenzhen University Medical School, Shenzhen University, Shenzhen 518060, China

**Keywords:** RNase P, Chromatin assembly, DNA damage response, Genome stability, Tumorigenesis

## Abstract

Ribonuclease P (RNase P) was first described in the 1970’s as an endoribonuclease acting in the maturation of precursor transfer RNAs (tRNAs). More recent studies, however, have uncovered non-canonical roles for RNase P and its components. Here, we review the recent progress of its involvement in chromatin assembly, DNA damage response, and maintenance of genome stability with implications in tumorigenesis. The possibility of RNase P as a therapeutic target in cancer is also discussed.

## Introduction

As a ribozyme, ribonuclease P (RNase P) was initially discovered in *Escherichia coli* [1,2], which is participated in the maturation of transfer RNAs (tRNAs), 4.5S RNA, riboswitches, and messenger RNAs (mRNAs) [3–7]. This ribozyme processes the 5′ leader sequences of precursor tRNAs (pre-tRNAs) and leaves a mature 5′ terminus and 3′ hydroxyl group on the leader sequence by hydrolyzing the phosphodiester bond within the RNA backbone [8,9]. As its activity relies on Mg^2+^ ions, RNase P is classed as a metalloenzyme [10]. In human, RNase P from nucleus is composed of up to ten protein subunits and a catalytic RNA component, H1 [11]. Remarkably, RNase P has several common protein subunits with two other eukaryotic ribonucleoprotein enzymes, *i.e.*, ribonuclease mitochondrial RNA processing (RNase MRP) and telomerase, which have distinct catalytic RNA components [12,13]. RNase MRP processes precursor ribosomal RNAs (rRNAs) by cleavage at specific sites [14,15], while telomerase protects telomeres from shortening during successive cell divisions by the addition of repetitive sequences to chromosome ends [16,17]. The sharing of subunits suggests that the protein subunits may be able to separate from the holoenzymes and participate in other intracellular processes.

Recent research progress reveals that RNase P and its components interact with various RNA substrates and get involved in transcription regulation or genome homeostasis maintenance, thus, have several non-canonical functions. First of all, purified RNase P from HeLa cells is proved to promote the maturation of mRNAs transcribed from RNA polymerase II (RNA Pol II) [4]. Protein components of RNase P are also reported to bind to RNA polymerase I (RNA Pol I) subunits and rRNA gene loci, interfering with the processing of rRNAs, although the precise mechanism remains unclear [18–21]. Moreover, RNase P RNA component regulates the transcription of 5S rRNAs transcribed from RNA polymerase III (RNA Pol III) [22]. Several protein components of RNase P bind to H3.3 (a histone H3 variant that regulates chromatin organization), compromising chromatin assembly and genome stabilization [23,24]. The genome suffers from continual assaults, both external and intrinsic, which can result in vast number of DNA damages, including DNA double-strand breaks (DSBs), which results in completely breakages of two DNA strands. Such damage triggers cells to mobilize and coordinate cellular activities to repair the damaged DNA in order to maintain genomic stability and integrity [25,26]. Intriguingly, two unique subunits of RNase P, RPP21 and RPP29, are found to be recruited at DSB sites, promoting homologous recombination (HR)-mediated DNA repair [27]. Furthermore, subunits of yeast RNase P are also involved in stabilizing the RNA component of telomerase, thereby protecting the ends of chromosomes [28].

Here, we summarize recent findings about RNase P assembly as well as non-canonical functions of RNase P and its components which are involved in a variety of cellular processes including chromatin assembly, DNA damage response, genome stability maintenance, and tumorigenesis. Furthermore, we discuss its potential in therapeutic drug development.

## Canonical function of RNase P

**Figure 1:**
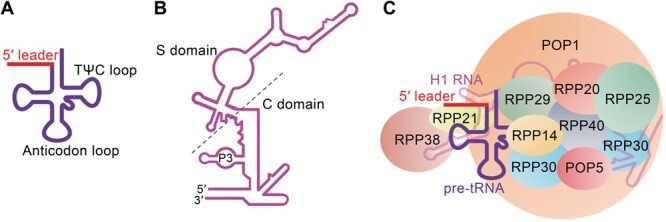
Schematic showing stabilization of RNase P during the processing of pre-tRNA 5′ leader sequences **A**. pre-tRNA model with 5′ leader (indicated in red). **B**. Model of human H1 RNA. The C and S domains are divided by the dashed line. **C**. Ten RPPs wrap around H1 RNA and pre-tRNA to ensure pre-tRNA processing. C, catalytic; S, specificity; tRNA, transfer RNA; RNase P, ribonuclease P; pre-tRNA, precursor tRNA; RPP, RNase P protein.

As a large ribozyme, RNase P consists of one single catalytic RNA [RNase P RNA (RPR)] and numerous protein subunits [RNase P proteins (RPPs)]; however, the exact number of protein components in RNase P depends on the domain of life (bacteria, archaea, or eukaryote) [29]. RNase P is facilitated by its structural organization to precisely function within a highly intricate cellular environment [30]. As in other ribozymes, maintenance of the correct folding of RPRs depends on divalent ions, especially Mg^2+^, which are also vital for the catalytic activities, including its ability to interact with and cleave substrates [10,31–34]. Cryo-electron microscopy (cryo-EM) has allowed us to further understand the process by which RNase P recognizes the 5′ leader sequence of pre-tRNA. A dual anchoring mechanism prevalent in bacterial, archaeal, and eukaryotic RNase Ps allows the TΨC loop of pre-tRNA (**Figure 1**A) to fit into the space between the catalytic (C) domain and specificity (S) domain of RNase P (Figure 1B), ensuring high specificity of pre-tRNA processing [35–40]. Simultaneously, core components of human RNase P, H1 RNA and POP1, anchor the pre-tRNA by measuring a conserved distance of 12 bp from the cleavage site of the pre-tRNA, enabling the rest of RPPs to correctly locate the pre-tRNA active site and carry out its activity (Figure 1C) [11,41]. This structure contributes to the anchoring of pre-tRNAs as well as to their cleavage [11,41,42].

Compositional variations of the spatial organization and counts of RPPs in all domains of life may affect substrate recognition and catalytic activity [43,44]. In bacteria and archaea, the RNase Ps consist of RNA components such as M1 RNA (*E. coli*), P RNA (*Bacillus subtilis* and *Thermotoga maritima*), and RPR (*Methanocaldococcus jannaschii*) ranging from 252 nt to 382 nt, while protein components are composed mainly of a small basic protein (RnpA/C5/P) in bacteria or a protein complex (consisting of five proteins, Pop5, Rpp30, Rpp29, Rpp21, and L7Ae) in archaea [35,36,45–47]. Bacteria P protein appears to stabilize the P RNA via an arginine-rich motif, thereby promoting substrate recognition [35,48–50]. In yeast, the RNA component (Rpr1; 369 nt) is accompanied by nine protein subunits, Pop8, Pop6, Rpr2, Pop7/Rpp2, Pop4, Rpp1, Pop3, Pop1, and Pop5 [39]. Genetic studies have demonstrated intricate interactions between the RPPs; the loss of a single RPP results in decreased cleavage efficiency, indicating that all subunits are indispensable [51]. Similarly, human nuclear RNase P H1 RNA (341 nt) is also accompanied by up to ten protein subunits, POP1, POP5, RPP20, RPP25, RPP30, RPP40, RPP38, RPP14, RPP29, and RPP21 [11]. RNase P shares seven subunits with RNase MRP, so far. RPP21, RPP29, and RPP14 appear to be unrelated with RNase MRP, and thus, are likely to be unique to RNase P [52–54]. H1 RNA acts as a scaffold for the protein subunits to stabilize the ribozyme, meanwhile, POP1, one of the eukaryotic-specific and the largest protein components of RNase P, acts as a molecular glue to support the RNA–protein interaction network [55–57].

In all species, RPRs are core to the catalytic activities of RNase Ps, while the presence of variable amount of RPPs is instrumental to ensure the catalytic function of the RPR [2]. As shown in Figure 1C, the C-terminus of POP1 stabilizes H1 RNA by wrapping around it, while the N-terminus of POP1 connects POP5, together contributing to the stabilization of the 5′ leader sequence of pre-tRNA [57]. POP1 serves as a molecular scaffold, interacting with each component of RNase P [11,35]. In addition, the RPP20–RPP25 heterodimer harbors a P3-binding subdomain of the C domain, through which the catalytic activity of RNase P is influenced [52,58]. The RPP25-like protein (RPP25L) can substitute for RPP25 in pre-tRNA processing activities [59]. Moreover, a heterotrimeric RPP29–RPP21–RPP38 complex coordinates the C and S domains of H1 RNA [35,60], while the RPP40 subunit contributes to the architectural core of the H1 RNA C domain [35]. In *Saccharomyces cerevisiae*, Rpp1 was reported to facilitate the processing of pre-tRNAs at 5′ and 3′ ends, as well as rRNAs at multiple sites [61]. However, it is not clear if its human ortholog RPP30 has a similar function.

In general, RPR has more complicated secondary structure and a greater level of activity *in vitro* from bacteria to archaea and eukaryotes despite indistinctive changes of length [62–66]. RNase Ps from yeast and humans exhibit the greatest level of complexity, with both consisting of an RNA component combined with multiple protein subunits [2,11]. Comparison of bacterial, archaeal, and eukaryotic RNase Ps by cryo-EM shows that bacterial RNase P has the most concise composition, while archaeal RNase P appears to be a transitional form, combining features of both bacterial and eukaryotic RNase Ps [11,36]. Structurally and functionally, it seems that RNase P in eukaryotes may have evolved from the bacterial and archaeal ones, acquiring more protein subunits as a structural substitution for a more condense RNA [2,43,67,68]. This “evolution” also seems to endow RNase P and its components with more ambiguous functions and biological significance.

## Ambiguous roles of RNase P in regulation of non-tRNA substrates

In the “evolved” eukaryotic RNase P, as the proportion of protein components becomes higher and the spatial structure becomes more complex [43,67,68], RNase P and its subunits display the potential to participate in more biological processes, given that RNase P recognizes substrates through a dual anchoring mechanism [36–40] and that within gene transcription its RNA products may become substrates for RNase P. On the other hand, the different properties of the increased protein subunits also have the potential to affect the processing of substrates [48–50]. Currently, RNase P and its components process not only pre-tRNAs, but also other RNA substrates through three dimensions: H1 RNA only, multi-protein complexes, and holoenzyme RNase P.

### H1 RNA

The catalytic core component of RNase P, H1 RNA, exhibits catalytic function on other RNA substrates besides pre-tRNAs, particularly small non-coding RNAs (sncRNAs) transcribed by RNA Pol III and Pol I [19,20]. When the S domain of H1 RNA is paired with its complementary DNA (cDNA) sequence and disrupted by RNase H, the transcription of sncRNAs mediated by RNA Pol III including 5S rRNA, 7SL RNA, and tRNA, as well as the transcription of rRNA mediated by RNA Pol I, is reduced, and the correct cleavage of pre-tRNAs is also impaired [19,20]. H1 RNA is as well recruited to the 5S rRNA gene locus and affects the formation of the initiation complex of RNA Pol III [22].

### Protein components

Currently, the protein components of RNase P appear in two main forms in the studies of processing of other RNA substrates, namely with or without H1 RNA. *In vivo*, the regulation of substrates is mediated mainly by the combination of several protein subunits, while *in vitro* several protein subunits are assembled and then form mini-RNase P with H1 RNA.

However, mini-RNase P, consisting of RPP21, RPP29, and H1 RNA, has only a cleavage effect on pre-tRNAs, but not on the sncRNAs transcribed by RNA Pol III [19].

Recent studies revealed that RPP20 and RPP25, with the feature of dual binding capacity for DNA and RNA, occupied tRNA and rRNA genetic loci, as RPP20 and RPP25 are also conserved proteins from Alba-like superfamily related to RNA metabolism [18–21]. For protein-only assembly, with a consequential decrease of RPP20 and RPP21, knockdown of *RPP25* in HeLa cells inhibited the assembly of the transcription initiation complex by repressing RNA Pol III on transcribing 5S rRNA, but not pre-tRNA cleavage [20,22]. Transcriptional complex formation of RNA Pol III was hypothesized to be stabilized by the formation of a RPP20–RPP25 heterodimer or by interaction with H1 RNA, thereby facilitating the formation of active chromatin [22,69]. Transcription of sncRNAs such as 5S rRNA, 7SL RNA, and tRNA were inhibited when RPP20, RPP21, RPP29, or RPP40 was removed in HeLa cells, respectively [19]. Moreover, RPP14, RPP21, RPP30, and RPP40 were found to bind to tRNA and 5S rRNA gene loci, and RPP20, RPP25, RPP21, RPP29, RPP30, and RPP38 were also found to bind to RNA Pol I-transcribed gene loci, including 5.8S rRNA, 18S rRNA, and 28S rRNA gene loci in a cell cycle-dependent manner [20,22]. Among them, RPP20, RPP25, RPP21, and RPP29 were essential for effective ribosomal DNA (rDNA) transcription by RNA Pol I, while each of them incorporating into transcription sites independently [20]. In yeast, knockdown of *Pop7* (the yeast ortholog of human *RPP20*) resulted in pre-tRNA accumulation as well as 35S rRNA processing defects [70].

In addition, mRNAs containing *N*^6^-methyladenine (m^6^A) modifications are also substrates of RPP complex composed of RPP20, RPP25, and POP1 [71,72]. The adaptor protein HRSP12 connects the m^6^A reader protein YTHDF2 to RPPs, enabling RPPs to process YTHDF2-bound transcripts [71,72]. Since m^6^A is committed to maintaining the quality and further translation of mRNAs by recruiting m^6^A-binding proteins [73,74], collectively, the ability of RPPs and RPR to regulate RNA Pol I/Pol II/Pol III-mediated transcription directly or indirectly points to a possible broader role for RNase P and its components in maintaining the cellular transcriptome.

### RNase P

Beyond the cleavage of pre-tRNAs, RNase P as a holoenzyme is also contributed to the regulation of other RNA substrates, such as human metastasis-associated lung adenocarcinoma transcript 1 (*MALAT1*) mRNA transcribed by RNA Pol II and its downstream tRNA-like structure, *MALAT1*-associated small cytoplasmic RNA (mascRNA). Purified RNase P from HeLa cells regulates the processing of the 3′ end of *MALAT1* mRNA, facilitated by a recently identified antisense transcript *TALAM1* at the same locus, resulting in mature *MALAT1* mRNA accumulation [4,75,76].

RNase P can also degrade a targeted mRNA when mediated by an external guide sequence (EGS), and a tRNA-like structure can be formed when this RNA fragment interacts with the targeted mRNA [77–79]. For example, EGSs have been designed to guide purified HeLa RNase P to process *ICP4* mRNA, encoding an activator for herpes simplex virus 1 (HSV1) gene transcription, via attaching EGS to *ICP4* mRNA complementary sequence [77,80]. Similar approach has been employed to knock down the expression of the human Werner syndrome RecQ like helicase (*WRN*) in cancer cell lines, resulting in severe inhibition of cell survival [81,82]. Thus, incorporating RNase P cleavage activity with EGSs may provide a therapeutic approach for HSV1, other viral infections, and cancers.

Taken together, RNase P acts as a holoenzyme that regulates mRNAs transcribed by RNA Pol II [75], and H1 RNA alone also affects the transcription activities of RNA Pol I and Pol III on rRNAs and sncRNAs [20,22]. The multi-protein complexes, mainly anchored by RPP25, promote the transcription activities of RNA Pol I and Pol III, and multiple protein subunits are recruited to RNA Pol I/Pol III-transcribed loci in a cell cycle-dependent pattern [18–22]. In addition, multi-protein complexes process m^6^A-modified mRNAs and therefore participate in maintaining the quality of the cellular transcriptome [71,72].

Since RPP20–RPP25 harbors the C domain of H1 RNA, while both RPP25 and the S domain of H1 RNA are deeply involved in the processing of rRNAs and sncRNAs, H1 RNA may be harbored by the multi-protein complexes in the non-tRNA substrate processing. However, it remains unknown whether these biological processes are accomplished by the holoenzyme RNase P or individual subunits.

## Beyond RNA: maintenance of genome stability

As we have learned, RNase P interferes with gene transcription and regulates various types of RNA substrates in addition to processing pre-tRNAs [44,83]. In terms of its effect on rRNA processing [20], this capability intersects with that of its companion RNase MRP, and the interchangeability and interpenetration properties between subunits offer unlimited potential for RNase P. Some subunits of RNase P have recently been indicated to be involved in biological processes beyond RNA, and with its impact on the transcriptome [19], so here we will discuss whether it also affects genome assembly and genome stability through transcriptional regulation or other mechanisms.

### Effects of RNase P on genome assembly

RNase P shares certain common protein subunits with two other vital eukaryotic ribonucleoprotein enzymes, RNase MRP and telomerase, while their catalytic RNA components are unique [84,85], leading to a crosstalk of the multi-functions between these enzymes. Common subunits of RNase P, RNase MRP, and telomerase are involved in regulating genome stability. For example, in *S. cerevisiae*, Pop1, Pop7, and Pop6 are required to stabilize the RNA component of telomerase (*TLC1*) and transport *TLC1* into nucleus, safeguarding telomerase formation [28,86] and thereby maintaining telomere length, as shortening of telomere has been considered as a hallmark of cancer [87]. Further investigation is required to determine whether these protein subunits also play a role in telomere maintenance in mammalian cells.

Recent studies have demonstrated that RNase P and its components interface with other fundamental elements of the genome. Two unique components of RNase P, RPP29 and RPP21, along with POP1, were proved to interact with histone H3.3, thus hindering the chromatin remodeling activity in actively transcribed genes and in turn compromising chromatin assembly and genome stabilization [23,24]. Studies by both Shastrula et al. and Newhart et al. showed that the N-terminus of RPP29 interacted with the H3.3, thereby repressing H3.3 binding into actively transcribed rRNA, mRNA, and tRNA gene loci; however, the precise underlying mechanism remains elusive [23,24]. Knocking down *RPP29* also decreased H3K9me3 and H3K27me3 (epigenetic marks associated with transcriptional regulation), supporting a role for RPP29 in regulating H3.3 recruitment to actively transcribed genes [23]. Meanwhile, POP1 and RPP21 but not the RNA components of RNase P/MRP were also found to inhibit the rate of transcription when they were recruited to histone H3.3-associated active transcriptional regions [24]. Additionally, RPP25 and RPP20 (common components of RNase P/MRP) as well as RPP29 (a unique component of RNase P) were reported to bind to the chromatin of active rDNA gene loci which is dependent on cell cycle, and the following recruitments of RNase P at 5S rRNA and tRNA gene loci further inhibited the assembly of genome [20]. Despite the capability of RPP25 and RPP20 to heterodimerize in RNase P/MRP, binding of these molecules to chromatin occurred independently [20].

Furthermore, a previous study in *Drosophila* showed that mutation of Rpp30 (subunit of RNase P in *Drosophila*) in ovaries activated DNA damage checkpoint that is dependent on checkpoint kinase 2 (Chk2), resulting in reduced levels of H3K9me3 in the chromatin that around tRNA genes, along with replication fork stalling and altered transcription of the PIWI-interacting RNA (piRNA) cluster [88]. In addition, in the prefrontal cortex (PFC) of autism patients, higher levels of H3K4me3 in the *RPP25* gene resulted in decreased *RPP25* transcripts [89]. By this way, it is possible that RPP25 or a multi-protein complex plays a role in pathogenicity through its own post-translational modifications in chromatin. These findings suggest the existence of unknown transcriptomic regulatory functions of RNase P components that may affect genome stability.

### Effects of RNase P on genome stability

The functions of RNase P components relate not only to chromatin assembly but also, more widely, to the regulation of overall genome stability. The human genome is continuously exposed to a large scale of exogenous and endogenous damaging factors [90]. DSBs represent the most aggressive and lethal type of DNA damage, as the paired DNA strand leaves one unrepaired breakage, causing cell death [25,26]. A series of repairs is initiated following cell damage. DSBs are repaired mainly by the following mechanisms: (1) non-homologous end-joining (NHEJ); (2) HR, and (3) microhomology-mediated end joining (MMEJ). Both HR repair and MMEJ are relied on poly(ADP-ribose) polymerase 1 (PARP1) which acts as an essential DNA damage response factor to promote further signaling through ADP-ribosylation with or without binding to poly(ADP-ribose) polymer (PAR) [91,92]. During the cell cycle, NHEJ is mediated by KU70/80 to rapidly repair DSBs, where makes it an error-prone process; however, unlike NHEJ and MMEJ, HR repair is a high-fidelity process. HR repair uses homologous chromosomes as templates for repair and occurs in late S and G2 phases, facilitated by DNA break-end trimming (carried out by a series of nucleases) as well as inter-strand invasion by RAD51 [91].

**Figure 2:**
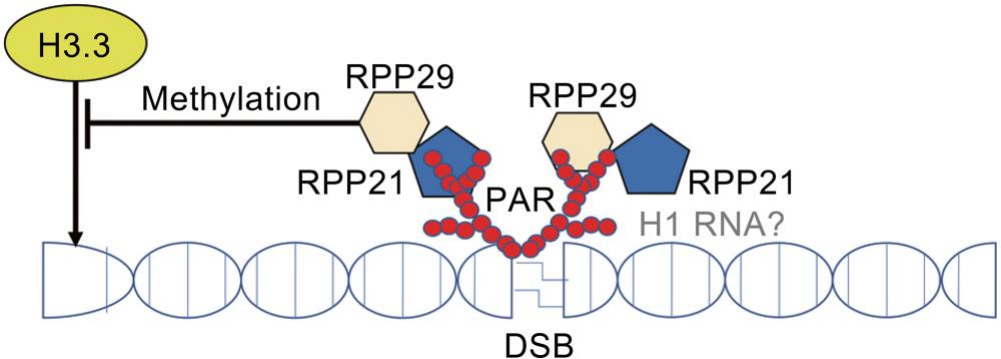
RNase P is recruited to DNA DSBs and represses H3.3 recruitment to actively transcribed genes RPP21 and RPP29 are recruited to DNA DSBs by binding to PAR moieties, while H1 RNA may provide a potential platform for the further recruitment of downstream HR repair proteins. RPP29 represses H3.3 incorporation into actively transcribed genes by regulating H3K9me3 and H3K27me3. DSB, double-strand break; PAR, poly(ADP-ribose) polymer; HR, homologous recombination.

Intriguingly, two unique components of human RNase P, RPP21 and RPP29, are recruited to DNA damage sites apart from their functions in RNase P [27]. These subunits are implicated in the regulation of HR-mediated DNA repair, thus maintaining genome stability. According to bioinformatics analysis and *in vitro* PAR binding assay, RPP29 and RPP21 possess PAR-binding motifs and are proved to be able to bind PAR moieties *in vitro* [27]. Furthermore, RPP21 and RPP29 are recruited to UV laser-induced DNA damage sites, which are dependent on the binding to PARP and the presence of the H1 RNA, but how RPP21 and RPP29 interact with H1 RNA is yet to investigate. In this process, PAR provides a platform for the further recruitment of downstream repair factors (**Figure 2**) [27]. Besides, H1 RNA coordinates with RPP21 and RPP29 to perform cleavage activity *in vitro*, shedding light on the potential of H1 RNA as a platform for the recruitment of RPPs or HR factors to DNA damage sites, which may also be inseparable from the relationship with the PARP family [27,93].

In addition, genome stability can be regulated directly or indirectly by mitochondrial RNase P (mt-RNase P) [94], which is only composed of three protein subunits *(i.e.*, MRPP1, MRPP2, and MRPP3) encoded by the nuclear genome [95]. Aberrant mt-RNase P activity on mitochondrial tRNAs affects mRNA levels of mitochondrial proteins [96–99], where abnormal RNA metabolism reduces ATP production and increases reactive oxygen species production, which eventually triggers apoptosis and promotes the maintenance of genome stability [99,100]. Deacetylation of MRPP2 has also been shown to protect cells under oxidative stress, thereby maintaining genome stability [101]. The missense mutation of *MRPP2* not only leads to malfunction of tRNA maturation, but also causes deregulated dehydrogenase/reductase activity triggering HSD10 disease [102]. That is, mt-RNase P maintains genome stability directly through post-translational modifications (PTMs) of its subunits or indirectly by compromising its classical tRNA processing capacity to affect other biological processes.

Overall, RNase P and its components have non-canonical functions that affect genome assembly and genome stability through a variety of mechanisms in addition to regulating non-tRNA substrates (**Table 1**). RNase P and its components modulate mRNAs, sncRNAx, and rRNAs transcribed by RNA Pol I, Pol II, and Pol III in three dimensions: (1) specific domain of H1 RNA contributes to RNA Pol I/Pol III-mediated transcription [19,20], (2) different multi-protein complexes modulate different RNA substrates in different mechanisms [19,20,22,71,72], and (3) holoenzyme RNase P exhibits cleavage activities with proper mediators [4,75–79]. Additionally, the potential effect of multiple RNase P subunits on genome length by affecting telomerase stability comes into play first [86]. Secondly, RNase P components impede chromatin assembly by binding to histone H3.3 and by PTMs [23,24], which inhibits the rate of transcription and thus affects genome stability. Besides, the effect of RNase P components on chromatin assembly is in a cell cycle-dependent manner [20], although the exact mechanism is still unknown. More importantly, the recruitment of two unique subunits of RNase P, RPP29 and RPP21, to the DNA damage sites is direct evidence for the involvement of RNase P components in the maintenance of genome stability [27]. Also, mt-RNase P contributes to the maintenance of genome stability [96–102].

**Table 1:** Non-canonical functions of RNase P and its components

Non-canonical function	Description	RNase P component	Refs.
mRNA regulation	Mediating cleavage of mRNA with tRNA-like structure and m^6^A modification	RNase P; the complex composed of RPP20, RPP25, and POP1	[4,71,72,75,76]
	Incorporating RNase P cleavage activity with EGSs to catalyze targeted mRNA	RNase P	[77–79]
sncRNA regulation	Modulating the transcription activity of RNA Pol III; inhibiting initiation complex of sncRNA transcription	H1 RNA; the complex composed of RPP20, RPP25, RPP21, RPP29, and RPP40	[19,22]
rRNA regulation	Modulating the transcription activity of RNA Pol I; binding to rRNA gene loci	H1 RNA; the complex composed of RPP20, RPP25, RPP21, RPP29, RPP30, and RPP38	[20]
Genome assembly	Maintaining telomere length	The complex composed of Pop1, Pop7, and Pop6	[28,86]
	Hindering chromatin assembly	The complex composed of RPP21, RPP29, POP1, RPP25, and RPP20; RPP30	[23,24,88]
Genome stability	Participating in HR-mediated DNA repair	The RPP21–RPP29 complex	[27]
	mt-RNase P protects cells under oxidative stress and regulates dehydrogenase/reductase activity	mt-RNase P	[96–102]

*Note*: RNase P, ribonuclease P; RPP, RNase P protein; mRNA, messenger RNA; sncRNA, small non-coding RNA; rRNA, ribosomal RNA; tRNA, transfer RNA; ESG, external guide sequence; RNA Pol I, RNA polymerase I; RNA Pol II, RNA polymerase II; RNA Pol III, RNA polymerase III; HR, homologous recombination; mt-RNase P, mitochondrial RNase P.

## RNase P and tumorigenesis

The aforementioned studies suggest that RNase P and its components have non-canonical functions in regulating chromatin assembly and DNA damage responses, in addition to processing basic biomolecules such as pre-tRNA and other RNA substrates. Thus, RNase P also has the potential to crucially affect the maintenance of genome stability and modulation of cellular growth. Consistent with this notion, high levels of H1 RNA were identified in colorectal cancer (CRC), inducing epithelial–mesenchymal transition (EMT) and contributing to CRC metastasis through inhibition of β-III tubulin ubiquitination [103]. Moreover, in gastric cancer (GC), H1 RNA overexpression was observed to promote tumor progression by inhibiting the expression of the negative cell cycle checkpoint regulator P21, and highly expressed H1 RNA in GC was associated with poor prognosis [104]. Overexpression of H1 RNA also facilitated tumor progression in non-small cell lung cancer, acute myeloid leukemia, and breast cancer by suppressing the expression of the microRNAs (miRNAs) miR-326, miR-330-5p, and miR-122, respectively [105–107]. In cervical cancer, *RPP25* was negatively regulated by miR-3127-5p and miR-338-3p, while up-regulation of *RPP25* promoted cell migration, invasion, and EMT [108,109]. Additionally, RPP30 was reportedly highly correlated with glioblastoma (GBM); low expression of RPP30 may be a trigger for GBM, serving as an independent prognostic factor for GBM [110].

Although the components of RNase P seem to occupy a significant position in human cancers, the molecular mechanisms underlying their contributions to tumorigenesis remain obscure. There is an urgent need to investigate how RNase P contributes to tumorigenesis so as to develop effective therapeutic strategies. Accordingly, we have summarized the alterations in RNase P components in an array of cancers from The Cancer Genome Atlas (**Table 2**). We found amplification of at least one RNase P component coding gene in 12 cancer cohorts with more than 250 patients each. Amplification of *POP1* and *RPP29* was frequently observed, such as in ovarian epithelial tumors and endometrial cancer; these RNase P subunits may have particular roles in the promotion of tumorigenesis and could subsequently be developed as potential prognostic markers. In addition, *POP1* mutations were identified at high frequencies in esophagogastric cancer, endometrial cancer, melanoma, and CRC and were especially prominent in endometrial cancer (7.00% of 586 cases). Overall, this dataset supports the notion that alterations in RNase P components are significantly associated with tumorigenesis.

**Table 2:** Gene alterations in RNase P components in human cancers

Cancer type	No. of cases	H1 RNA	*POP1*	*POP5*	*RPP14*	*RPP20*	*RPP21*	*RPP25*	*RPP29*	*RPP30*	*RPP38*	*RPP40*
Ovarian epithelial tumor	584	–	7.88% (A)	–	–	–	3.42% (A)	–	17.81% (A)	–	–	6.68% (A)
Esophagogastric cancer	622	–	3.86% (A), 3.86% (M)	–	–	6.11% (A)	–	–	8.68% (A)	–	–	–
Endometrial cancer	586	–	7.00% (M), 4.27% (A)	–	–	–	–	–	9.39% (A)	–	–	–
Bladder cancer	411	–	9.00% (A)	–	–	–	–	–	5.11% (A)	–	–	–
Melanoma	444	–	6.53% (M)	–	–	–	–	–	–	–	–	3.60% (A)
Breast cancer	1084	–	9.50% (A)	–	–	–	–	–	–	–	–	–
Non-small cell lung cancer	1053	–	3.42% (A)	–	–	–	–	–	3.51% (A)	–	–	–
Sarcoma	255	–	–	–	–	–	–	–	5.49% (A)	–	–	–
Hepatobiliary cancer	372	–	6.99% (A)	–	–	–	–	–	–	–	–	3.23% (A)
Prostate cancer	494	–	7.89% (A)	–	–	–	–	–	–	–	–	–
Head and neck cancer	523	–	3.25% (A)	–	–	4.02% (A)	–	–	–	–	–	–
Colorectal cancer	594	–	3.37% (M)	–	–	–	–	–	–	–	–	–

*Note*: Data were obtained from www.cbioportal.org in September 2022. Gene alterations with a frequency > 3% and patient cohort size > 250 were included. A, amplification; M, mutation; −, not available.

## Future perspectives

The structure of RNase P from three domains of life has been reported through cryo-EM research, enabling us to explore what actually happens in this ribozyme. It is worth highlighting that the role of protein components in RNase P is progressively being clarified. Initially known for a specific role in pre-tRNA processing, RNase P and its components are now understood to play a role in chromatin stability, transcription, and DNA damage response, prompting us to wonder how RNase P regulates these subunits to perform different biological functions.

When contributing to the regulation of non-tRNA substrates, RNase P from HeLa cells promotes the maturation of mRNAs, and tRNA-like structures or mediators such as adaptor and reader proteins are responsible for RNase P cleavage activity [71–77]. Secondly, RPP20, RPP21, RPP25, RPP29, and RPP40 promote sncRNA processing [18,21], RPP20, RPP25, RPP21, and RPP29 promote rRNA processing [19,20], and H1 RNA devotes to regulating RNA Pol I/Pol III-mediated transcription. In this case, H1 RNA may be harbored by multi-protein complexes during the processing of non-tRNA substrates, which may come to a point that protein components of RNase P still cooperate with H1 RNA even multiple protein subunits form a functional complex in other biological processes. Production rates for both tRNA and rRNA are modulated by multi-protein complexes in different processes, thus compromising the transcriptional activities of RNA Pol I and Pol III, the pre-tRNA cleavage activity, and even the genome assembly [70]. Since H1 RNA is also transcribed by RNA Pol III [111], it is possible that the functional complexes of RNase P components not only affect other genes and transcription products, but also regulate themselves.

From another perspective, the three vital ribonucleoprotein enzymes in eukaryotes, RNase P, RNase MRP, and telomerase, share certain common protein subunits, while the catalytic RNA components attach them to their biological functions [84,85]. Human RNase MRP shares seven subunits with RNase P, including POP1, POP5, RPP20, RPP25, RPP30, RPP38, and RPP40 [52], while the RPP20–RPP25 heterodimer interacts with the P3 domains of RNase MRP and RNase P RNA [58,112,113]. In addition, the Pop6–Pop7 heterodimer (the yeast homolog of RPP20–RPP25) was reported to bind to and stabilize the P3-like region of *TLC1* [28,86], suggesting that RPPs act in a noncatalytic manner to regulate other RNA substrates or RNA–protein complexes. Notably, the P3 regions of the RNA components of yeast RNase P, RNase MRP, and telomerase are mechanically interchangeable [84,114], suggesting that these three ribonucleoprotein enzymes may be subject to underlying interdependent mechanisms of regulation.

In addition, the composition of RPPs endows them with unique properties. Bacterial P proteins contain arginine-rich motifs that bind to both P RNA and pre-tRNA, although intrinsically disordered regions (IDRs) rich in arginine/lysine motifs give the protein structural flexibility and polymorphism, which is the characteristic of RNA-binding proteins and a feature of liquid–liquid phase separation (LLPS) [115–118]. Human POP1 and RPP29 also have conserved arginine-rich IDRs that allow RNase P to recognize pre-tRNAs, while RNase MRP is potentially capable of processing different RNA substrates [112,113,119]. It suggests that polymorphic RPPs may have the ability to control the spatial and temporal distribution of RNA ligands through LLPS and thus, maintain the distribution of transcriptional regulators for the regulation of gene expression.

The identification of potentially new components of RNase P provides additional insight into the roles of this enzyme in various biological processes. For example, further interactors for RPPs have been identified by yeast two-hybrid screening and pull-down assays. Such interactors include heat shock protein 27 (HSP27), LIM domain-containing protein 1 (LIMD1), and opacity-associated protein 2/Exosome component 8 (OIP2/EXOSC8) [120]. As a 3′→5′ exoribonuclease, OIP2 participates in the processing of pre-tRNA at the 3′ terminus alongside RPP14 [121], while HSP27 acts as an ATP-independent chaperone to stimulate RNase P catalytic activity, although it is not essential for RNase P activity [120]. The precise mechanisms by which these cofactors modulate RNase P assembly and enzymatic activity remain to be determined. The newly found RPP25L which can substitute for RPP25 in pre-tRNA processing also provides a novel perspective for the compatibility of RNase P [59]. The existence of other cofactors regulating the non-canonical functions of RNase P also remains to be more thoroughly explored.

In light of the aforementioned studies, we hypothesize how RNase P and its components regulate multiple functions simultaneously: (1) formation of multi-protein complexes, *e.g.*, by adjusting the amount of common subunits in different complexes to regulate rRNA transcription, which thus feedbacks on the efficiencies of genome assembly and pre-tRNA processing; (2) regulation of RNase P or its multi-protein complexes by other functional proteins, *e.g.*, regulation by PARP family or RNA Pol I and Pol III complexes that bind to subunits, or formation of small compartments through LLPS mediated by RNA ligands. Despite the fact that the basic function and structure of RNase P have been identified, its biological functions are undoubtedly complicated. Since it is not one single subunit of RNase P to catalyze other RNA substrates and participate in other biological processes, where the underlying regulatory mechanisms are not clear, we assume that RNase P is more likely to perform multiple biological functions as a dynamic complex through changes in the content of different subunits or in combination with different functional units, which in turn maintains transcriptome and genome homeostasis. The regulatory relationships between its subunits and other elements of the genome need to be further explored.

The non-canonical roles identified for RNase P and its components in transcription, chromatin assembly, DNA damage response, and tumorigenesis show that RNase P plays an important role in genome stability maintenance. Utilizing RNase P activity via EGSs as a guide to cleave target *ICP4* mRNA is now a novel therapeutic approach for HSV1 [77,80], providing an alternative therapeutic choice for viral infections. However, the underlying molecular mechanisms of how RNase P functions in various biological processes await elucidation. A previous study showed that RNase P activity increased in a PARP-dependent manner upon DNA damage in U2OS cells [27], suggesting that RNase P inhibitors and PARP inhibitors could be used as combination therapies in cancer therapies. Such studies will serve as a foundation for the development of potential drugs to target RNase P.

## CRediT author statement

**Peipei Wang:** Visualization, Methodology, Writing – review & editing. **Juntao Lin:** Writing – original draft, Investigation. **Xiangyang Zheng:** Conceptualization, Data curation, Supervision. **Xingzhi Xu:** Conceptualization, Data curation, Supervision. All authors have read and approved the final manuscript.

## Competing interests

The authors have declared no competing interests.
